# The correlation of acupuncture and moxibustion points for the treatment of diminished ovarian reserve and premature ovarian failure: A systematic review based on complex network

**DOI:** 10.1097/MD.0000000000040185

**Published:** 2024-11-01

**Authors:** Zhihong Yang, Zhou Zhu, Xiaofang Yang, Wei Zhang, Panbi Chen, Linming Jin, Qiuping Luo, Yuqiong Meng, Jiamin Liu

**Affiliations:** a College of Acupuncture and Tuina, Guizhou University of Traditional Chinese Medicine, Guiyang, China; b First Affiliated Hospital of Guizhou University of Traditional Chinese Medicine, Guiyang, China; c Department of Acupuncture and Massage Rehabilitation, The First Affiliated Hospital of Hunan University of Chinese Medicine, Changsha, China.

**Keywords:** acupuncture, complex network technology, diminished ovarian reserve, moxibustion, premature ovarian failure

## Abstract

**Background:**

Infertility rates have risen in recent years, with diminished ovarian reserve (DOR) affecting 10% of infertile women, accounting for approximately 20% of ovarian disorders. This highlights the importance of exploring effective treatments for DOR. This study investigates acupoint compatibility rules in acupuncture and moxibustion therapies for DOR and premature ovarian failure (POF) using complex network analysis. It also provides insights into acupuncture and moxibustion approaches for managing these conditions.

**Methods:**

Clinical studies on acupuncture and moxibustion treatments for DOR and POF were retrieved from 5 databases, including China National Knowledge Infrastructure, SinoMed, Wanfang, CQVIP, and PubMed, covering the period from January 1, 2007, to September 15, 2024. Literature was screened based on inclusion and exclusion criteria to build a comprehensive database of acupuncture and moxibustion prescriptions for these conditions. Association rule analysis was performed using IBM SPSS Modeler 18.0, and complex network analysis was conducted with Gephi 0.9.2 software.

**Results:**

A total of 70 articles and 67 acupoints from 70 prescriptions were analyzed, with 665 occurrences of these acupoints. The most frequently used acupoint was Guanyuan (RN4), followed by Sanyinjiao (SP6), Zusanli (ST36), Shenque (RN8), Bladder 23 (BL23), and Zigong (EX-CA1). These acupoints were primarily related to the Ren (RN) and Bladder (BL) meridians. Jiaohui points, dorsal Yu points, and 5 Shu points were the most commonly utilized specific points. The combination of Sanyinjiao (SP6) and Guanyuan (RN4) had the strongest association. Complex network analysis revealed a small-world network effect, with 24 core acupoints, including Guanyuan (RN4) and Zusanli (ST36), serving as key nodes.

**Conclusion:**

Acupuncture treatments for DOR and POF primarily focus on the abdomen and lower back regions. Specific acupoints, such as rendezvous points, are frequently combined with distantly located points. These combinations are guided by therapeutic principles aimed at nourishing the kidney, strengthening the spleen, regulating liver qi, calming the heart, and promoting blood circulation.

## 1. Introduction

Diminished ovarian reserve (DOR) is a condition where women of reproductive age show decreased number and quality of follicular cells in the ovary before the age of 40 years. Menstrual disorder, infertility, and reduced sinus follicle count are the primary clinical manifestations of DOR, possibly accompanied by perimenopausal symptoms, such as vaginal dryness, hot flashes, night sweats, and mood changes.^[1,2]^ According to statistical reports, 10% of female infertility issues are caused by DOR.^[3]^

The DOR cases are on a rise due to the accelerated pace of modern life, increase in economic and social pressures, deterioration of environmental pollution, and delay in childbearing age. In addition, youthfulness shows an increasing trend, with a young age of onset. The decline in ovarian function is a gradual process. The absence of timely and effective interventions for DOR may lead to ovarian atrophy within 1 to 6 years, eventually developing into premature ovarian failure (POF).^[4]^ Hence, improving the ovarian reserve function can delay POF and diminish infertility. Regarding the open policy of 3 children, late marriage, and late childbirth, improving the ovarian reserve function of women planning pregnancies is significant for better fertility. At present, the main methods of western medicine for treating decreased ovarian reserve function are hormone replacement therapy, ovulation induction therapy, immunosuppressive therapy, etc. Although they can restore ovarian function in a short period of time, they have big side effects, are prone to recurrence, and must be taken for a long period of time,^[5]^ which is not easy for some patients to accept. Through reviewing the literature, we found that the efficacy of acupuncture to improve the ovarian reserve function is accurate, and acupuncture in Chinese medicine emphasizes the identification of evidence, holistic treatment, which can achieve the purpose of both symptomatic and curative treatment.^[6]^

Overall, traditional Chinese medicine (TCM) treatment regulates multiple levels, targets, systems, and links. The efficacy of acupuncture in improving ovarian reserve function is definite. Moreover, clinical studies have proven the scientific and clinical usefulness of acupuncture DOR and POF treatments.^[7,8]^ Modern complex network technology is not only necessary but also beneficial for determining the optimal combination of acupoints for specific diseases, such as the need for acupuncture in clinical practices and the development of acupuncture from traditional empirical medicine to evidence-based medicine. Therefore, the present study explored the characteristics and association law of acupoints for treating DOR and POF, providing a relatively standard prescription for acupuncture and moxibustion treatments.

## 2. Methods

### 2.1. Search methods

Five Chinese databases, including the China National Knowledge Infrastructure, SinoMed, Wanfang, and CQVIP, and an English database, such as PubMed, were searched for studies on DOR and POF treatments using acupuncture and moxibustion from January 1, 2007 to September 15, 2024. The languages were restricted to English and Chinese. All search terms were determined using MeSH terms and free words. The selected search terms were as follows: DOR, ovarian failure, POF, acupuncture, acupuncture therapy, acupuncture treatment, ear acupunctures, acupoint catgut embedding, abdominal acupuncture, electroacupuncture, moxibustion, and randomized clinical trial.

### 2.2. Inclusion criteria

(1) Types of studies: the randomized clinical trial aimed to evaluate the effectiveness of acupuncture or moxibustion treatments for DOR and POF.
(2) Participants: participants in the included studies were diagnosed with DOR and POF.
(3) Intervention: acupuncture or moxibustion treatments were used for DOR and POF, and other traditional Chinese and Western medicine treatment methods could be combined, such as Chinese herbs, Western medicine, or other physical therapies.
(4) Clear description of the specific acupoints was provided.
(5) Processes that have clear efficacy in the conclusion of the experiment and are amenable to all efficacy evaluations during the course of the experiment.

### 2.3. Exclusion criteria

(1) Nonclinical randomized controlled trials, including but not limited to individual cases or recipe cases, reviews, conference papers, animal experimental research, clinical literature without a control group, and Masters and Doctoral degree thesis.
(2) Acupuncture was not used as the main therapy in the treatment groups.
(3) Literature with no original data or only abstracts.
(4) Repeatedly published literature.
(5) Minimum sample size was <20 in each group.^[9]^

### 2.4. Database establishment and data processing

#### 2.4.1. Database establishment

The data on acupuncture and moxibustion treatments for DOR and POF was established using Microsoft Excel software. Searches were conducted independently by 2 researchers. The articles were screened according to the inclusion and exclusion criteria. The articles that did not meet the inclusion criteria were excluded after reading their titles and abstracts, while those that met the inclusion criteria were selected and read. In addition, the selected articles were independently extracted and summarized.

#### 2.4.2. Standardization of acupoint names

The acupoints included in the study were standardized according to the “Name and Positioning of Meridian Points (GB/T 12346-2021).”

#### 2.4.3. Standardization of acupoint prescriptions

In all, 61 articles were included in the study, with 61 valid prescriptions.

#### 2.4.4. Data analysis

##### 2.4.4.1. Association rule analysis

The association rule refers to the regularity of simultaneous occurrence between 2 or more variables. An association rule is an expression of the form X→Y, where X and Y are disjointed item sets. The expression represents the relationship between the occurrence of item sets X and Y. The strength of the association rule can be measured in terms of its support, confidence, and lift. Support determines how often a rule applies to a given data set and measures whether an association between X and Y happens by chance. Confidence establishes how frequently items in Y appear in transactions that contain X and represents the reliability of the association. Lift is the ratio between observed and expected support when X and Y are independent.^[10]^

In the present study, the association rule analysis was performed using the Apriori algorithm via IBM SPSS modeler18.0 software (support and confidence degrees were set to >15% and >80%, respectively). In addition, acupoints and meridian patterns of acupuncture and moxibustion were obtained for DOR and POF.

##### 2.4.4.2. Complex network analysis

The association rule analysis fails to inspect the overall pattern of using acupoints together as it assesses the association between a limited number of item sets. Complex networks describe the system structure primarily through network angles to explore the universal law. The complex association network was constructed by considering the acupoints as nodes and the compatibility combination between acupoints as edges. The complex networks exhibit the internal characteristics and properties of acupoints directly by demonstrating the connections between them. K-core and modularity analyses were performed using Gephi0.9.2 software to study the acupoint compatibility of acupuncture and moxibustion for DOR and POF.

## 3. Results

### 3.1. Literature screening results

From 105 articles retrieved in both English and Chinese, 70 were selected based on inclusion and exclusion criteria. These articles provided 70 acupoint prescriptions across multiple meridians, involving 67 different acupoints. These acupoints were used a total of 665 times (Guidelines Flow Diagram and Table 1).

**Table 1:** Summary of included studies.

Author/year	Country	Sample size	Study design	Method
Deng Lixing (2019)^[11]^	China	90	Randomised controlled trial	Moxibustion, acupoint catgut embedding
Zhu Lijuan et al (2018)^[12]^	China	60	Randomised controlled trial	Medicinal herb, moxibustion
Li Fang et al (2020)^[13]^	China	60	Randomised controlled trial	Medicinal herb, acupuncture, moxibustion
Yi Shangguo (2012)^[14]^	China	76	Randomised controlled trial	Medicinal herb, moxibustion
Chen Jun et al (2019)^[15]^	China	90	Randomised controlled trial	Medicinal herb, acupoint catgut embedding
Kang Jianhua et al (2019)^[16]^	China	40	Randomised controlled trial	Medicinal herb, moxibustion
Gao Buchan et al (2019)^[17]^	China	63	Randomised controlled trial	Medicinal herb, acupoint catgut embedding
Qi Ding et al (2021)^[18]^	China	89	Randomised controlled trial	Electroacupuncture, acupoint injection
Yu Xiwei et al (2019)^[19]^	China	90	Randomised controlled trial	Acupuncture, moxibustion
Zhou Li et al (2015)^[20]^	China	30	Randomised controlled trial	Acupuncture, electroacupuncture, warming needle moxibustion
Zhang Linyun et al (2021)^[21]^	China	60	Randomised controlled trial	Pricking-cupping bloodletting, moxibustion
Bian Xinhui et al (2016)^[22]^	China	60	Randomised controlled trial	Acupoint catgut embedding
Chen Min et al (2017)^[23]^	China	18	Randomised controlled trial	Acupoint catgut embedding
Tang Haixia (2011)^[24]^	China	84	Randomised controlled trial	Acupoint catgut embedding
Ji Jiaping (2019)^[25]^	China	60	Randomised controlled trial	Medicinal herb, moxibustion
Yang Lijuan et al (2019)^[26]^	China	66	Randomised controlled trial	Medicinal herb, acupuncture
Song Meiling et al (2019)^[27]^	China	80	Randomised controlled trial	Acupuncture, acupoint catgut embedding
Wang Hui et al (2020)^[28]^	China	50	Randomised controlled trial	Acupuncture
Wang Yu et al (2018)^[29]^	China	96	Randomised controlled trial	Medicinal herb, moxibustion
Niu Yongqin et al (2017)^[30]^	China	90	Randomised controlled trial	Acupuncture, moxibustion, medicinal herb
Yang Xiaohong et al (2008)^[31]^	China	60	Randomised controlled trial	Acupuncture, moxibustion
Lai Yuqin et al (2018)^[32]^	China	186	Randomised controlled trial	Acupuncture, warming needle moxibustion
Liu Kaiya et al (2018)^[33]^	China	78	Randomised controlled trial	Medicinal herb, electroacupuncture
Lai Yuqin et al (2017)^[34]^	China	116	Randomised controlled trial	Medicinal herb, electroacupuncture
Zhang Lixia et al (2016)^[35]^	China	46	Randomised controlled trial	Medicinal herb, acupuncture
Zhang Xiaohong et al (2015)^[36]^	China	60	Randomised controlled trial	Medicinal herb, acupuncture
Teng Yan et al (2020)^[37]^	China	40	Randomised controlled trial	Medicinal herb, acupuncture, acupoint catgut embedding
Pan Li et al (2014)^[38]^	China	60	Randomised controlled trial	Medicinal herb, electroacupuncture
Xing Hongmei et al (2008)^[39]^	China	55	Randomised controlled trial	Medicinal herb, acupuncture
Wu Song et al (2018)^[40]^	China	50	Randomised controlled trial	Medicinal herb, moxibustion
Wei Yanping et al (2010)^[41]^	China	132	Randomised controlled trial	Warming needle moxibustion, ginger insulation
Bian Qinhua et al (2022)^[42]^	China	40	Randomised controlled trial	Medicinal herb, moxibustion
Hu Jin et al (2022)^[43]^	China	60	Randomised controlled trial	Medicinal herb, acupuncture
Zhou RouZhi et al (2022)^[44]^	China	60	Randomised controlled trial	Warming needle moxibustion
Teng Jing et al (2017)^[45]^	China	56	Randomised controlled trial	Acupuncture
Xiao Qingfeng et al (2015)^[46]^	China	40	Randomised controlled trial	Warming needle moxibustion
Wu Xiaomin et al (2018)^[47]^	China	68	Randomised controlled trial	Acupuncture
Pang Miaomiao et al (2017)^[48]^	China	60	Randomised controlled trial	Acupuncture, warming needle moxibustion
Wan Niya et al (2021)^[49]^	China	130	Randomised controlled trial	Acupuncture
Li Deping et al (2018)^[50]^	China	30	Randomised controlled trial	Medicinal herb, warming needle moxibustion
Tan Xuming et al (2017)^[51]^	China	44	Randomised controlled trial	Acupuncture, warming needle moxibustion
Chi Yanyan (2019)^[52]^	China	126	Randomised controlled trial	Acupoint catgut embedding
Zhang Yi et al (2017)^[53]^	China	120	Randomised controlled trial	Medicinal herb, acupuncture
Zeng Chunyan (2018)^[54]^	China	90	Randomised controlled trial	Medicinal herb, acupuncture, ginger insulations
Lin Lingli (2021)^[55]^	China	40	Randomised controlled trial	Acupuncture
Wang Qin et al (2020)^[56]^	China	60	Randomised controlled trial	Medicinal herb, acupoint catgut embedding
Xu Baixing et al (2022)^[57]^	China	60	Randomised controlled trial	Acupuncture, moxibustion
Huang Hai et al (2020)^[58]^	China	80	Randomised controlled trial	Moxibustion, acupoint catgut embedding
Yang Xin et al (2021)^[59]^	China	74	Randomised controlled trial	Moxibustion
Wang Qingwei et al (2021)^[60]^	China	66	Randomised controlled trial	Moxibustion
Zhang Yue (2021)^[61]^	China	60	Randomised controlled trial	Moxibustion
Cui Leilei et al (2022)^[62]^	China	80	Randomised controlled trial	Medicinal herb, moxibustion
Shi Yange et al (2021)^[63]^	China	105	Randomised controlled trial	Medicinal herb, acupoint catgut embedding
Xie Ying et al (2020)^[64]^	China	60	Randomised controlled trial	Medicinal herb, moxibustion
Feng Xiao-Ling et al (2020)^[65]^	China	75	Randomised controlled trial	Acupuncture, moxibustion
Peng Yanli et al (2022)^[66]^	China	92	Randomised controlled trial	Electroacupuncture, medicinal herb
LI Yu (2021)^[67]^	China	122	Randomised controlled trial	Acupuncture, medicinal herb
Deng Meilan et al (2022)^[68]^	China	80	Randomised controlled trial	Medicinal herb, warming needle moxibustion
Zhou Rouzhi et al (2022)^[44]^	China	60	Randomised controlled trial	Medicinal herb, acupoint catgut embedding
Du Xiaona et al (2022)^[69]^	China	80	Randomised controlled trial	Acupuncture
Li Miaomiao et al (2022)^[70]^	China	60	Randomised controlled trial	Acupuncture
Wang Yin et al (2022)^[71]^	China	80	Randomised controlled trial	Acupuncture
Yuan Shixin et al (2022)^[72]^	China	60	Randomised controlled trial	Warming needle moxibustion
Huang Yufeng et al (2023)^[73]^	China	100	Randomised controlled trial	Acupuncture, moxibustion
Zhang Xiuhong et al (2023)^[74]^	China	80	Randomised controlled trial	Medicinal herb, acupoint catgut embedding
Yang Likun et al (2023)^[75]^	China	104	Randomised controlled trial	Moxibustion, warming needle moxibustion
Ying Zheng et al (2015)^[76]^	China	240	Randomised controlled trial	Acupuncture
Le Yang et al (2021)^[77]^	China	338	Randomised controlled trial	Electroacupuncture
Anlun Yi et al (2021)^[78]^	China	119	Randomised controlled trial	Electroacupuncture
Huanfang Xu et al (2021)^[79]^	China	120	Randomised controlled trial	Medicinal herb, acupuncture

### 3.2. Frequency of acupoints and meridians in DOR and POF treatments

In the treatment of DOR and POF across 70 articles, a total of 67 acupoints were used, amounting to 665 instances of usage. The top 5 most frequently used acupoints were Guanyuan (RN4, 62 times), Sanyinjiao (SP6, 58 times), Zusanli (ST36, 49 times), Shenque (RN8, 46 times), and Zigong (EX-CA1, 33 times). Additionally, the frequency and number of acupoints were analyzed across different meridians. The top 3 meridians in terms of usage frequency were the Ren Meridian (ReN, 162 times), the Bladder Meridian (BL, 119 times), and the Spleen Meridian (SP, 98 times). When ranked by the number of acupoints used, the top 3 were the Ren Meridian (ReN, 12 acupoint), the Bladder Meridian (BL, 11 acupoint), and the Kidney Meridian (KI, 9 acupoint) (Table 2).

**Table 2:** Frequency of acupoints and meridians in DOR and POF treatments.

Meridians	Acupoint number	Frequency	Acupoints (frequency)
ReN	12	162	Guanyuan (CV4, 62), Qihai (CV6, 33), Zhongji (CV3, 32), Zhongwan (CV12, 16), Shenque (CV8, 6), Xiawan (CV10, 3), Yinjiao (CV7, 3), Huiyin (CV1, 1), Danzhong (CV17, 2), Shangwan (CV13, 2), RN2–1, RN9–1.
Bladder	11	119	Shenshu (BL23, 46), Pishu (BL20, 24), Ciliao (BL32, 16), Ganshu (BL18, 18), Shenshu (BL17, 6), Zhongliao (BL33, 2), Feishu (BL13, 1), Shangliao (BL31, 1), Weishu (BL21, 3), Xialiao (BL34, 1), Xinshu (BL15, 1).
Spleen	7	98	Sanyinjiao (SP6, 58), Xuehai (SP10, 23), Diji (SP8, 6), Yinlingquan (SP9, 5), Gongsun (SP4, 4), Daheng (SP15, 1), Fushe (SP13, 1).
Stomach	6	90	Zusanli (ST36, 49), Guilai. (ST29, 17), Tianshu (ST25, 14), Shuidao (ST28, 4), Qichong (ST30, 4), Fenglong (ST40, 2).
Kidney	9	59	Taixi (KI3, 27), Siman (KI14, 7), Fuliu (KI7, 3), Huangshu (KI16, 3), Shiguan (KI18, 2), Yindu (KI19, 1), Yongquan (KI1, 1), Zhaohai (KI6, 2), Dahe (KI12, 13).
DU	6	43	Mingmen (DU4, 16), Baihui (DU20, 11), Shenting (DU24, 6), Yaoyangguan (DU3, 6), Dazhui (DU14, 2), Yaoshu (DU2, 2).
Extraordinary points	3	36	Zigong (EX-CA1, 33), Shiqizhui (EX-B7, 2), Sishencong (EX-HN1, 1).
Liver	3	24	Taichong (LR3, 22), Ligou (LR5, 1), Ququan (LR8, 1).
Others	2	13	Luanchao (9), Qixue (4).
Pericardium	2	7	Neiguan (PC6, 6), Ximen (PC4, 1)
Gallbladder	2	7	Benshen (GB13, 5), Yanglingquan (GB34, 2).
Lung large	1	3	Lieque (LU7, 3).
Intestine	1	2	Hegu (LI4, 2).
Heart	2	2	Yinxi (HT6, 1), Shenmen (HT7, 1).

DOR = diminished ovarian reserve; POF = premature ovarian failure.

### 3.3. Frequency of specific acupoints in DOR and POF treatments

**Figure 1.**
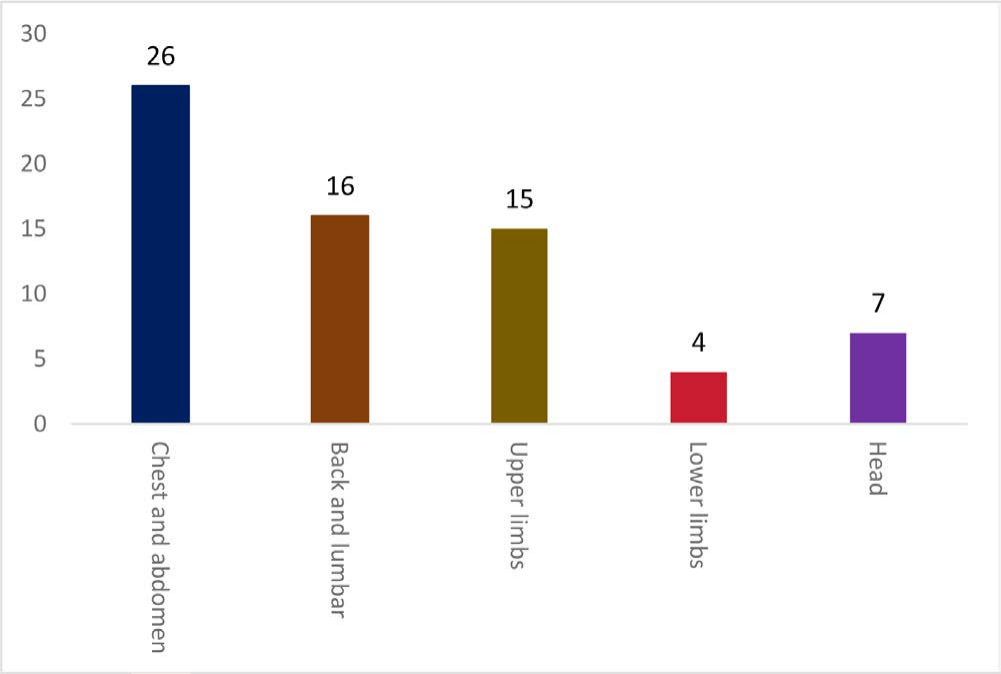
Frequency of acupoints in DOR and POF treatments in different body parts. DOR = diminished ovarian reserve; POF = premature ovarian failure.

The most frequently used specific acupoints are the rendezvous points (22), followed by the dorsal Yu points (7) and the 5 acupoints (6) (Fig. 1).

### 3.4. Frequency of acupoints in DOR and POF treatments in different body parts

**Figure 2.**
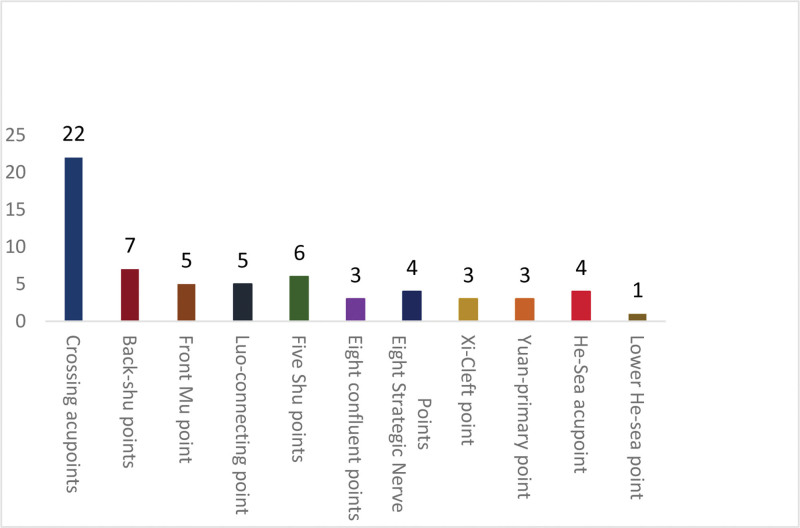
Frequency of specific acupoints in DOR and POF treatments. DOR = diminished ovarian reserve; POF = premature ovarian failure.

The acupoints were analyzed based on the locations of 70 specific points. The most frequently used were the thoracic and abdominal acupoints, with a total frequency of 26, followed by the dorsal and lumbar acupoints, lower limb acupoints, head acupoints, and upper limb acupoints, with frequencies of 16, 15, 7, and 4, respectively (Fig. 2).

### 3.5. Association rule analysis of acupuncture for DOR and POF

A total of 67 acupuncture points from 70 prescriptions were analyzed using association rules. The models were constructed with IBM SPSS Modeler 18.0 software, setting the support and confidence thresholds to >15% and >80%, respectively, using the Apriori algorithm.

**Figure 3.**
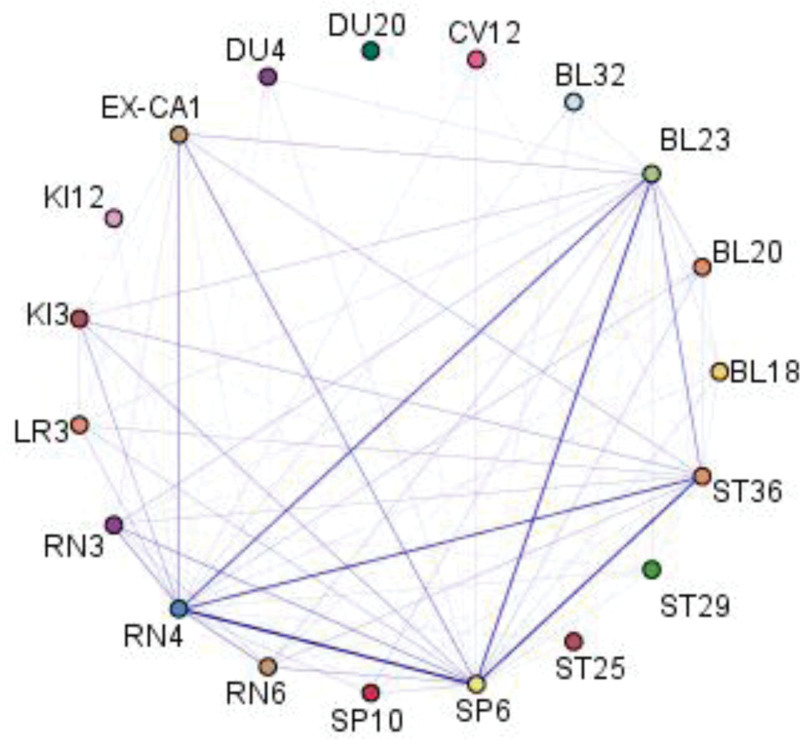
Acupuncture of DOR and POF prescription based on Association rule analysis. DOR = diminished ovarian reserve; POF = premature ovarian failure.

The schematic analysis of the association rules revealed that the pairwise combinations of Sanyinjiao (SP6) and Guanyuan (RN4), Guanyuan (RN4) and Sanyinjiao (SP6), as well as Shousanli (ST36) and Sanyinjiao (SP6), showed the highest support levels (Fig. 3). Table 3 presents the top 10 acupoint combinations with the highest support. The strongest association was found between Sanyinjiao (SP6) and Guanyuan (RN4), with support and confidence values of 84.286% and 84.746%, respectively. This indicates that at least 84.286% of prescriptions included both Sanyinjiao (SP6) and Guanyuan (RN4), while 84.746% of prescriptions containing Guanyuan (RN4) also included Sanyinjiao (SP6).

**Table 3:** Top 10 acupoint combinations in DOR and POF treatments.

Acupoint combinations	Support (%)	Confidence (%)
Sanyinjiao (SP6)→Guanyuan (RN4)	84.286	84.746
Guanyuan (RN4)→Sanyinjiao (SP6)	81.429	87.719
Sanyinjiao (SP6)→Zusanli (ST36)	68.571	91.667
Guanyuan (RN4)→Zusanli (ST36)	68.571	85.417
Sanyinjiao (SP6)→Shenshu (BL23)	64.286	93.333
Guanyuan (RN4)→Shenshu (BL23)	64.286	88.889
Sanyinjiao (SP6)→Zigong (EX-CA1)	47.143	93.939
Guanyuan (RN4)→Zigong (EX-CA1)	47.143	96.970
Guanyuan (RN4)→Qihai (RN6)	47.143	93.939
Sanyinjiao (SP6)→Zhongji (RN3)	44.286	93.548

DOR = diminished ovarian reserve; POF = premature ovarian failure.

### 3.6. Complex network analysis

#### 3.6.1. Basic parameters of complex network in acupuncture for DOR and POF

The acupoints and acupuncture prescriptions for DOR and POF were imported into Gephi 0.9.2 software in CSV format to calculate the basic parameters of the compatibility structure within the complex network. These parameters included the number of nodes and edges, average path length, network diameter, and average clustering coefficient. The results indicated that the acupuncture prescriptions for DOR and POF contained 67 nodes and 877 edges, with an average path length of 1.625, suggesting that on average, 1.625 acupoints were required to establish interconnections. Additionally, the network diameter was 3, implying an average of 3 acupoints in any given prescription. The clustering coefficient was 0.803 (Table 4).

**Table 4:** Basic parameters of acupoint collocation structures of acupuncture in DOR and POF treatments.

Attribute	Number
The number of nodes	67
The number of edges	877
The average path length	3
The network diameter	1.625
The average clustering coefficient	0.803

DOR = diminished ovarian reserve; POF = premature ovarian failure.

#### 3.6.2. Small-world network effects of acupoint collocation

As a characteristic of the complex network, the small-world network effect signifies that most of the nodes in the network are not connected but can be connected to each other through a few key nodes with special connections. Measuring a complex network with a small-world effect is determined by the ratio of the average path length to the average clustering coefficient, which is smaller than the ratio of a randomly set network. The results suggested that the ratios of the acupoint collocation network in acupuncture for DOR and POF were much smaller than the random network ratios, with a small-world network effect. It shows that acupoint combinations for DOR and POF were not random with certain acupoints but are composed under the linkage of core acupoints (Table 5).

**Table 5:** Comparison of acupuncture and moxibustion for DOR and POF with the random network.

Type	Average path length (L)	Average clustering coefficient (C)	Ratio (L/C)
Acupuncture and moxibustion for DOR and POF	1.63	0.80	2.04
Random network	1.84	0.05	36.80

DOR = diminished ovarian reserve; POF = premature ovarian failure.

#### 3.6.3. Analysis of acupoint nodes in acupuncture for DOR and POF

The importance of complex network nodes is often measured using degree, closeness centrality, and betweenness centrality, where the degree represents the number of acupoints connected to other acupoints. The larger the number of acupoints, the more acupoints connected with it and the stronger the compatibility, which are worth recommending in clinical practice. The closeness centrality reflects the average distance from a node to all other nodes. The greater the closeness centrality of a node, the more centrality that the node is located at the center of the complex network. The betweenness centrality indicates the distance and importance of either node to other nodes, and a larger value suggests the importance of the node.

The top 10 acupoints in the prescription of acupuncture and moxibustion for DOR and POF were selected according to the degree. The results showed that the top 10 acupoints had the same rankings with closeness and betweenness centralities (Table 6).

**Table 6:** Analysis of acupoint nodes in acupuncture for DOR and POF.

Acupoints	Degree	Closeness centrality	Betweenness centrality
Guanyuan (RN4)	63.00	0.96	196.10
Sanyinjiao (SP6)	60.00	0.92	128.66
Zusanli (ST36)	57.00	0.88	128.71
Qihai (RN6)	55.00	0.86	113.87
Shenshu (BL23)	52.00	0.83	68.10
Xuehai (SP10)	52.00	0.83	70.92
Zhongji (RN3)	50.00	0.80	78.43
Zigong (EX-CA1)	46.00	0.76	38.41
Zhongwan (CV12)	46.00	0.77	57.95
Taixi (KI3)	45.00	0.75	33.58

DOR = diminished ovarian reserve; POF = premature ovarian failure.

#### 3.6.4. K-core analysis of acupuncture for DOR and POF

**Figure 4.**
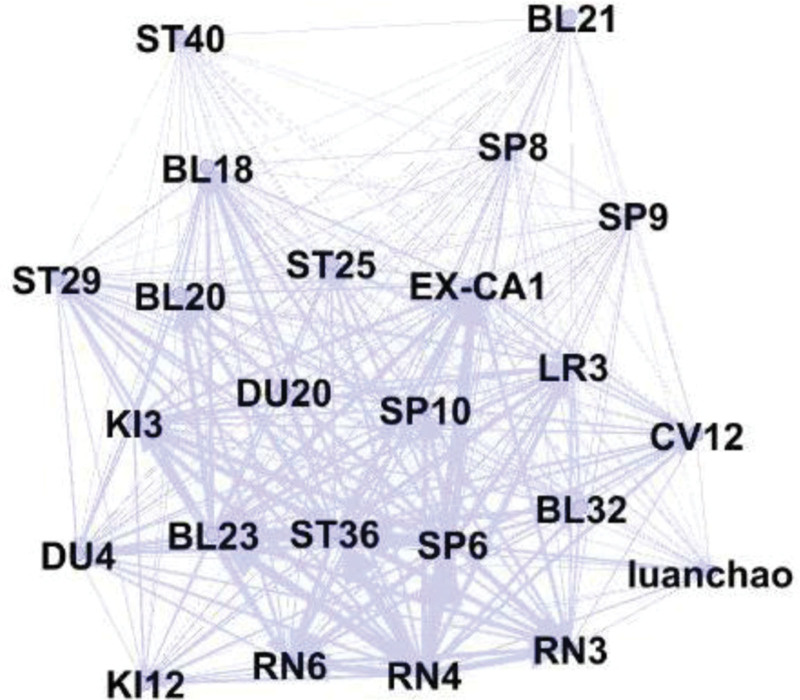
K-core analysis of acupuncture for DOR and POF. DOR = diminished ovarian reserve; POF = premature ovarian failure.

The core acupoints for acupuncture treatment of DOR and POF were identified using K-core analysis. The acupuncture prescription data for DOR and POF were imported into Gephi 0.9.2 software, and the Fruchterman–Reingold algorithm was applied to construct the model and perform K-core analysis. The results showed that the complex network graph disappeared when the K-core value reached 22, indicating that the maximum K-core of the network was 21, and 24 acupoints were identified as core nodes. These acupoints were the most frequently used across 70 acupuncture prescriptions and can be considered the primary acupoints for treating DOR and POF (Fig. 4).

#### 3.6.5. Community analysis of the complex network of acupoints in DOR and POF treatments

**Figure 5.**
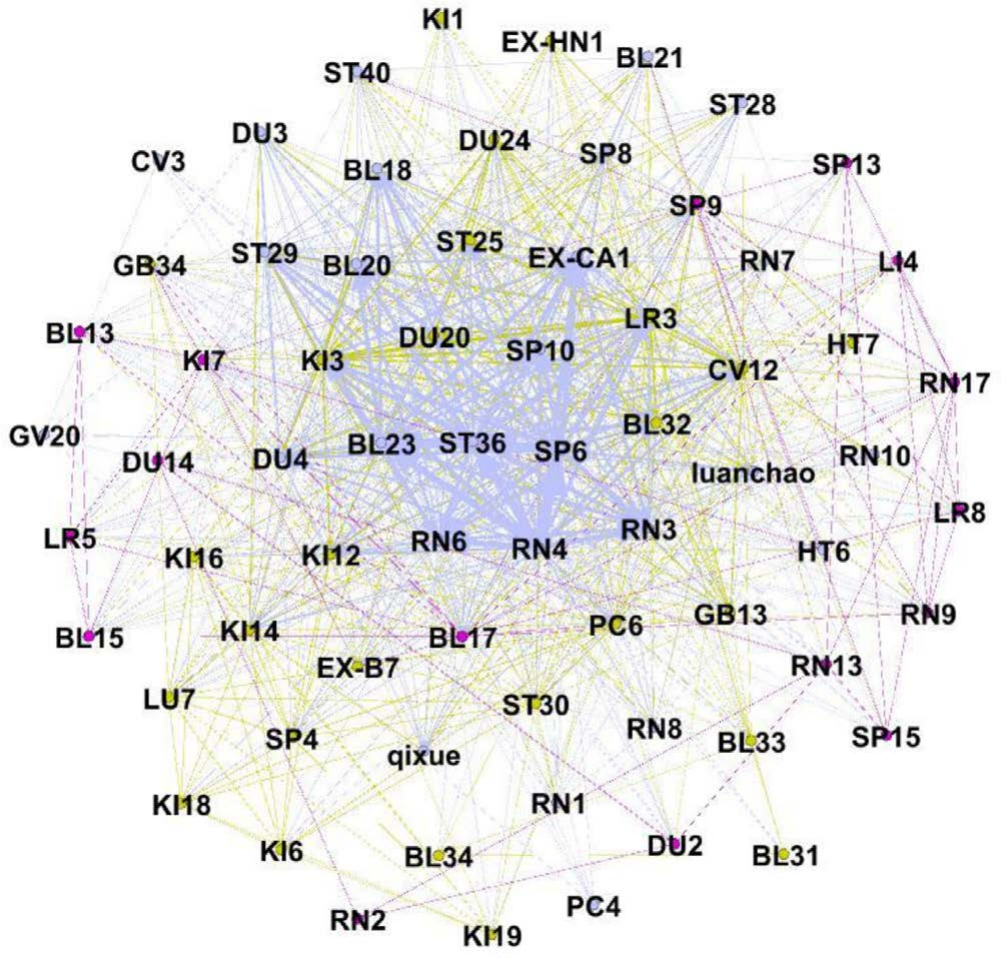
Association analysis of acupoints in DOR and POF treatments. DOR = diminished ovarian reserve; POF = premature ovarian failure.

Cluster analysis is a key component of complex network studies, and community analysis can visually represent the compatibility of acupuncture for DOR and POF. The results revealed 3 clusters in the network (Fig. 5). The blue cluster included acupoints such as Guanyuan (RN4), Sanyinjiao (SP6), and Shenque (BL23), indicating that acupuncture for DOR and POF frequently involves points from the Directing Vessel, Bladder Meridian, and Spleen Meridian. The yellow cluster comprised acupoints like Gongsun (SP4), Zhaohai (KI6), Yongquan (KI1), Qichong (ST30), Huiyin (RN1), and Ziwu (EX-CA1), suggesting a focus on both local and limb acupoints. This supports the acupuncture principles of “treating where the meridians pass” and “combining proximal and distal points.” Acupoints in the purple cluster were mainly Shang Guan (RN13) and Ququan (LR8), indicating that specific points, such as rendezvous points, transport points, and 5 Shu points, were commonly used in the acupuncture treatment of DOR and POF, where the meridians were abundant with qi and blood.

## 4. Discussion

Although DOR and POF are not nominally recorded in TCM, their clinical manifestations are classified into delayed menstruation, menstrual irregularities, scanty menstruation, and infertility. The TCM etiology includes the insufficient transformation of blood from the spleen and stomach, severe consumption of yin blood, and exhaustion of the blood source, and its pathogenesis involves kidney deficiency and dysfunction, with kidney deficiency being the most fundamental.^[80]^ TCM treatment for the disease is based on tonifying the kidney and replenishing its essence. Ancient experts selected invigorating spleen and supplementing qi (Jianpi Yiqi), dredging the liver to regulate qi (Shugan Liqi), nourishing the heart and mind (Tiaoyang Xinshen), and activating the blood and resolving stasis (Huoxue Huayu) as treatment methods according to syndrome differentiation. Clinical reports on acupuncture treatment for DOR and POF are increasing annually with remarkable clinical efficacy. Hence, it is a hot spot of research in recent years. In addition to simple acupuncture, electroacupuncture, catgut implantation at acupoints, moxibustion, and auricular acupuncture exist. Acupuncture and moxibustion therapies are based on meridians and collaterals in TCM, selecting acupoints for the condition through which acupoints are stimulated to run qi and blood, link meridians and internal organs, communicate with internal and external organs, regulate the whole body, and cure diseases. The principle of point selection is tonifying the kidney and nourishing its essence, dredging the liver to regulate qi, fortifying the spleen and nourishing blood (Jianpi Yangxue), and regulating and tonifying the Chong and Ren meridians (Tiaoli Chongren). It was found that acupuncture can improve oocyte quality, regulate the synthesis and conversion of DOR sex hormones, and also can regulate follicular development.^[81]^ These studies have confirmed the role of acupuncture for DOR.

The complex network technology was used to identify the acupoint selection based on 70 acupoint prescriptions in the clinic, providing a reference for acupuncture and moxibustion treatments for DOR and POF.

In this study, we collated 70 articles and analyzed 67 acupuncture prescriptions to determine the frequency of acupoint usage. The top 10 acupoints with the highest application frequency were Guanyuan (RN4), Sanyinjiao (SP6), Ashigaru Sanli (ST36), Shenque (BL23), Zigong (EX-CA1), Qihai (RN6), Zhongji (RN3), Taixi (KI3), Pishu (BL20), and Xuehai (SP10). The regularity of acupoint selection for acupuncture and moxibustion treatments for DOR and POF was analyzed as follows: (1) it is mostly associated with spleen–stomach and Chong–Ren. The spleen and stomach are the acquired foundations and the vital geneses of qi–blood. If the function of the spleen is strong in transporting and transforming food and drinks, it could produce essence, qi, blood, fluid, and humor. The thoroughfare and conception vessels originated from the uterus, and the blood filling of Chong meridian would be “Taichong pulse Sheng.” A blocked uterus can cause blood deficiency, leading to menstrual disorders; hemophthisis, leading to difficult pregnancy; and obstructed blood circulation leading to blood stasis. (2) The specific points is emphasized: in the selection of the alarm point of the conception vessel and transport point in the bladder meridian, the Qi of viscera and bowels were injected and converged. The results showed that the most frequent acupoint for DOR and POF was Guanyuan (RN4), which was the front-mu point of the small intestine, crossing points of the conception vessel, and 3 Yin meridians of the foot. It was compatible with Pishu (BL20) and Shenshu (BL23) of transport point, cultivating the Yuan and strengthening the root (Peiyuan Guben), fortifying the spleen and regulating the blood, and tonifying the kidney and regulating menstruation. (3) Local acupoints were closely associated with lesions. Regarding distribution, high-frequency acupoints, such as Zusanli (ST36) and Sanyinjiao (SP6), were the distant points, and Guanyuan (RN4), Qihai (RN6), and Zigong (EX-CA1) were distributed in the local, which reacted to the superior-inferior point combination of acupuncture.

According to the distribution of the meridians, the acupoints used in acupuncture and moxibustion treatments for DOR and POF were concentrated in the Ren meridians, Bladder meridian, and Kidney meridian. The ren meridians begins in the uterus, controlling reproduction, intersecting with all the Yin meridians in the body, and regulating the Qi and blood of the Yin meridians. Spleen meridian does not directly connect to the uterus, but it ascends along the medial side of the foot and tibia as well as the anteromedial side of the thigh to the lower abdomen to intersect with the conception vessel at Zhongji (RN3) and Guanyuan (RN4). The bladder meridian in the back is Yang, which runs through the back and lumbar and is connected to the 5 viscera and 6 bowels through the transport points. Besides, the kidney and liver meridians are high-frequency meridians of acupuncture and moxibustion treatments for DOR and POF, and it is the same as the medication principles of TCM.

The statistical results of the association rule analysis of acupoints showed that the acupoint frequency and high-frequency acupoint combinations were generally consistent with acupoints with a confidence degree ≥0.8. The most frequent acupoint combination was Guanyuan (RN4)–Sanyinjiao. It primarily reflects the acupoint combination principle of “distant–adjacent point combination.” Sanyinjiao (SP6) is the lower limb acupoint of the spleen meridian, which carries the essence and blood of the 3 Yin meridians of the liver, spleen, and kidney meridians. In addition, it is compatible with Guanyuan (RN4), regulating and tonifying the Chong and Ren meridians as well as soothing the liver and regulating the qi, harmonizing the qi, and moving the blood.

In summary, complex network technology analyzed and discussed the acupoint selection rules of acupuncture and moxibustion treatments for DOR and POF. Ancient experts focused on the visceral pattern syndrome differentiation in their clinical acupuncture points and were mostly associated with spleen–stomach and Chong–Ren. The application of specific acupoints was the crossing point, front-mu points, back-shu points, and 5 transport points. The local acupoints and lesions combination requires special attention, emphasizing the acupoint combination principle of “distant–adjacent point combination.” However, the present study had some limitations. There are few clinical research articles on acupuncture for DOR and POF, with only 70 articles included after repeated screening by 2 researchers. The selected articles lacked evidence-based classification, and only 9 of them incorporated evidence-based treatment. Chinese medicine should emphasize a holistic approach alongside evidence-based practices. By identifying relevant evidence, acupuncture treatment can be integrated with meridian circulation sites and associated internal organs. This approach can lead to more accurate acupoint prescriptions and enhance the application of acupuncture in reproductive health.

## Author contributions

**Conceptualization:** Zhihong Yang, Xiaofang Yang.

**Data curation:** Zhou Zhu.

**Funding acquisition:** Wei Zhang.

**Investigation:** Linming Jin, Qiuping Luo, Yuqiong Meng, Jiamin Liu.

**Methodology:** Zhou Zhu.

**Resources:** Panbi Chen.

**Software:** Qiuping Luo, Jiamin Liu.

**Supervision:** Xiaofang Yang.

**Validation:** Wei Zhang, Panbi Chen, Linming Jin.

**Writing – original draft:** Zhihong Yang, Zhou Zhu.

**Writing – review & editing:** Xiaofang Yang.

## References

[1] Practice Committee of the American Society for Reproductive Medicine. Testing and interpreting measures of ovarian reserve: a comittee opinion. Fertil Steril. 2015;103:e9–e17. 25585505 10.1016/j.fertnstert.2014.12.093

[2] Ong CL Thia EWH. Obstetrics and Gynecology. Berlin Heidelberg: Springer; 2015:445–466.

[3] Messerlian C Maclagan L Basso O. Infertility and the risk of adverse pregnancy outcomes: a systematic review and meta-analysis. Hum Reprod. 2013;28:125–37. 23042798 10.1093/humrep/des347

[4] Guzel Y Aba YA Yakin K Oktem O. Menstrual cycle characteristics of young females with occult primary ovarian insufficiency at initial diagnosis and one-year follow-up with serum amh level and antral follicle count. PLoS One. 2017;12:e0188334–14. 29176793 10.1371/journal.pone.0188334 PMC5703527

[5] Xia L Xia Y. Clinical research and progress of mechanism of action of acupuncture for premature ovarian failure in the last 20 years. Chin Acupunct Moxibustion. 2018;38:565–70. 10.13703/j.0255-2930.2018.05.031 29797923

[6] Wu S Yang R Feng X. Chinese medicine’s perception of decreased ovarian reserve function and the current status of acupuncture treatment. World J Integr Med. 2023;18:844–8.

[7] Xiu WC Gang WJ Jiao RM. Effect of acupuncture on outcomes of in vitro fertilisation: a scoping review. Chin J Integr Med. 2022;28:472–80. 34897592 10.1007/s11655-021-3459-6

[8] Wang Y Li Y Chen R Cui X Yu J Liu Z. Electroacupuncture for reproductive hormone levels in patients with diminished ovarian reserve: a prospective observational study. Acupunct Med. 2016;34:386–91. 27177929 10.1136/acupmed-2015-011014 PMC5099178

[9] Measures for the Administration of Drug Registration. China: Bulletin of the State Council of the People’s Republic of China; 2020:40–56.

[10] Leem J Jung W Kim Y Kim B Kim K. Exploring the combination and modular characteristics of herbs for alopecia treatment in traditional Chinese medicine: an association rule mining and network analysis study. BMC Complement Altern Med. 2018;18:204. 29973199 10.1186/s12906-018-2269-7 PMC6030800

[11] Deng LX. Effect of “Fuyang Du Meridian Moxibustion” combined with acupoint catgut embedding in the treatment of decreased ovarian reserve function. Contemp Chin Med. 2019;26:150–2.

[12] Xu Y Hu J Xiao S. Clinical study on the treatment of infertility due to decreased ovarian reserve function by combining the method of tonifying kidney and regulating menstruation with acupoint thermosensitive moxibustion. World Latest Med Inf Abstr (Contin Electron J). 2018;18:37–8.

[13] Li Fang YW Huang XJ. Treatment of kidney-tonifying and blood-activating Chinese medicine combined with acupuncture for decreased ovarian reserve function due to kidney-yin deficiency. Jilin Tradit Chin Med. 2020;40:1041–3.

[14] Yi S. The efficacy of the kidney-tonifying and spleen-strengthening prescription combined with moxibustion in the treatment of premature ovarian failure. Chin Med Guide. 2012;10:297–8.

[15] Tang H Ye L Wang F Li G Yu G Zhu X. Clinical study on the treatment of premature ovarian failure with Chinese medicine for tonifying kidney and soothing liver combined with acupoint catgut embedding. J Tradit Chin Med. 2019;47:112–5.

[16] Kang JH Zou H Tong JJ. Observation on the treatment of premature ovarian failure with the method of tonifying kidney and regulating the pulse combined with umbilical moxibustion. Guangming Chin Med. 2019;34:1802–4.

[17] Zhao R Yang F Liu Y. Danggui Shaoyao powder combined with acupoint catgut embedding for the treatment of liver depression and kidney deficiency type ovarian reserve function decline. Chin Med Inf. 2019;36:87–91.

[18] Zhang Y Wang B Hu X Li H. Clinical efficacy of electroacupuncture combined with placental polypeptide injection at Zusanli point in the treatment of decreased ovarian reserve function and its effect on sex hormone levels. Hebei Tradit Chin Med. 2021;43:117–120, 125.

[19] Yu X Zhu X Hua S. Clinical observation on umbilical acupuncture therapy combined with Hunyuan moxibustion in the treatment of premature ovarian failure. Guangming Chin Med. 2019;34:2035–7.

[20] Xia Y Lu J Wang Y. Clinical study on 30 cases of IVF-ET with decreased ovarian reserve treated with sequential acupuncture. Jiangsu Tradit Chin Med. 2015;47:58–60.

[21] Ye Y Lin W Hong X. Clinical study on acupoint catgut implantation for the treatment of infertility due to ovarian dysfunction. Chin J Health Standard Manag. 2021;12:121–4.

[22] Bian XH An Y Chen J. Clinical observation on acupoint catgut implantation for the treatment of premature ovarian failure. J Guangxi Univ Chin Med. 2016;19:19–21.

[23] Chen L Chen X Tian X. Clinical observation on the efficacy of acupoint catgut embedding in the treatment of premature ovarian failure. Sichuan Med. 2017;38:615–7.

[24] Tang H. Clinical observation on 40 cases of premature ovarian failure treated with Yishen Tiaochong decoction combined with moxibustion. Shanxi Tradit Chin Med. 2011;27:19–21.

[25] Ji JP. Observation on the efficacy of Yishen Kangsui Decoction combined with acupuncture in the treatment of 60 cases of ovarian reserve function decline due to kidney yin and yang deficiency. Health Read. 2019;33:178.

[26] Zhang J Zhang Z Xu S. Effect of acupuncture at Chong and Ren meridians on the improvement of sex hormones in patients with diminished ovarian reserve function. China Med Herald. 2019;16:157–61.

[27] Song ML Sheng XY. Effects of acupuncture on ovarian function and uterine ovarian blood flow index in patients with ovarian reserve dysfunction due to kidney deficiency and liver depression. Chin J Drugs Clin. 2019;19:2987–90.

[28] Chen X Xie B Jiang T Huang H. Effects of acupuncture combined with compound Zuogui capsule on low ovarian reserve function and its mechanism of action. World Chin Med. 2020;15:1346–50.

[29] Wang Y Yu HM. Observation on the efficacy of acupuncture combined with traditional Chinese and Western medicine in the treatment of premature ovarian failure. Shanghai J Acupunct. 2018;37:1042–6.

[30] Tian C Li J Liu H Liu Q. Analysis of the efficacy of acupuncture in treating decreased ovarian reserve function. J Clin Res Tradit Chin Med. 2017;9:11–4.

[31] Yang XH Lai XM Huang ZB. Clinical observation on 60 cases of premature ovarian failure treated with acupuncture and moxibustion. Sichuan Tradit Chin Med. 2008;26:106–7.

[32] Guo Q Xie L Yang L. 186 cases of ovulatory dysfunction with diminished ovarian reserve treated with traditional Chinese medicine and electroacupuncture. Psychol Doctor. 2018;24:128–9.

[33] Liu KY Qin QP. Observation on the effect of sequential cycles of traditional Chinese medicine combined with electroacupuncture in the treatment of decreased ovarian reserve function in patients with spleen and kidney deficiency. Chin Med Innov. 2018;15:65–8.

[34] Wei Y Ling P Long M. Clinical observation on 58 cases of premature ovarian failure treated with Chinese medicine cycle therapy combined with abdominal acupuncture. Hunan J Tradit Chin Med. 2017;33:52–3.

[35] Zhang LX He J. Clinical observation on the treatment of kidney deficiency type infertility with decreased ovarian reserve function by Chinese medicine cycle therapy combined with acupuncture. Zhejiang J Integr Tradit Chin West Med. 2016;26:1111–3.

[36] Zhang XH Deng LL. Clinical observation on the multi-channel application of traditional Chinese medicine in the treatment of decreased ovarian reserve function. Chin Med Innov. 2015;12:101–3.

[37] Teng Y Sun Y. Clinical efficacy of Zishen Yijing Huoxue decoction combined with acupuncture in the treatment of premature ovarian failure. Chin J Health Care Nutr. 2020;30:133.

[38] Lu Y Pang L Su Y Wen J. Clinical observation on 30 cases of ovarian reserve function decline treated with Zishen Yijing decoction combined with acupuncture. Chin J Ethnomedicine. 2014;23:29–30.

[39] Xing HM Wang R Yang H. Clinical study on the treatment of premature ovarian failure with a self-prescribed prescription for tonifying the kidney and strengthening the spleen combined with moxibustion. J Difficult Dis. 2008;7:101–2.

[40] Wu S Yan JG. Clinical observation on the treatment of premature ovarian failure with warm needling of Zusanli and Guanyuan points combined with ginger moxibustion of Baliao points. Chin Acupunct Moxibustion. 2018;38:1267–71. 10.13703/j.0255-2930.2018.12.004 30672213

[41] Zhao S Li Y Pan X Dong B. Clinical study on Zuogui Pills combined with moxibustion in the treatment of premature ovarian failure. Youjiang Med. 2010;38:168–70.

[42] Peng Q Peng H Zhu S Yin Z. Clinical observation on Zuogui Pills combined with acupuncture in the treatment of premature ovarian failure. Mod Distance Educ Tradit Chin Med. 2022;20:96–8.

[43] Hu J Yuan M. Effects of warm acupuncture combined with pelvic floor rehabilitation on patients with diminished ovarian reserve function. J Tradit Chin Med. 2022;33:653–5.

[44] Du J Yue A Yu W. Clinical observation on the treatment of premature ovarian failure with acupuncture for regulating menstruation and promoting pregnancy combined with Kuntai capsule. J Guangzhou Univ Tradit Chin Med. 2022;39:1071–7.

[45] Teng J Geng H Xu ZM. Clinical study on the treatment of premature ovarian failure of spleen and kidney deficiency type with Bushen Nuanchong decoction combined with acupuncture. Mod J Integr Tradit Chin West Med. 2017;26:2515–2517 + 2548.

[46] Xiao QF Fu B Zhou HF. Clinical observation on the treatment of premature ovarian failure with kidney-tonifying and blood-removing acupuncture. Hubei J Tradit Chin Med. 2015;37:58–9.

[47] Wu XM Qiu KS Li LY. Effects of Chong-Ren meridian acupuncture on ovarian function recovery and serum endocrine hormones in patients with premature ovarian failure. J Tradit Chin Med Pharm. 2018;24:80–2.

[48] Pang MM Hui JR Han H. Clinical efficacy of Tongluo Huoxue acupuncture in the treatment of premature ovarian failure. J Clin Mil Med. 2017;45:1028–30.

[49] Li S Sun HD Cui W. Clinical efficacy of warm acupuncture combined with Danggui Guizhi decoction in the treatment of premature ovarian failure and its effect on ovarian blood flow status. J Hubei Univ Tradit Chin Med. 2021;23:87–9.

[50] Li Z Gong X Xia W Li Q. Observation on the efficacy of warm acupuncture in treating premature ovarian failure of kidney yang deficiency type. J Tradit Chin Med. 2018;33:337–40.

[51] Fei L Yu C Lan S. Clinical observation on acupoint catgut embedding combined with western medicine for the treatment of premature ovarian failure. Hebei Tradit Chin Med. 2017;39:1089–92.

[52] Chi YY. Clinical observation on the treatment of premature ovarian failure with modified Yougui Pills, acupuncture and sequential therapy. World Chin Med. 2019;14:2474–8.

[53] Zhang Y Zhang Q. Clinical observation on the treatment of premature ovarian failure with acupuncture combined with traditional Chinese medicine. J Pract Chin Med. 2017;33:889–90.

[54] Zeng CY. Observation on the efficacy of acupuncture combined with Kuntai capsule in the treatment of premature ovarian failure. Chin J Health Standard Manag. 2018;9:110–1.

[55] Lin LL. Clinical analysis of the Bushen Shugan recipe combined with acupoint catgut embedding for the treatment of premature ovarian insufficiency. J Pract Chin Med. 2021;37:36–7.

[56] Huang W Gao F Liao H Yao M. Effects of abdominal acupuncture combined with Du-moxibustion on sex hormones and immune cell levels in patients with premature ovarian failure. J Yunnan Univ Tradit Chin Med. 2020;43:60–4.

[57] Sun X Luo L He X. Clinical study on the treatment of premature ovarian failure of spleen and kidney yang deficiency type by moxibustion combined with acupoint catgut embedding. New Chin Med. 2022;54:204–7.

[58] Li J Chen M He S. Study on the treatment of premature ovarian insufficiency with Kuntai capsule combined with moxibustion. Heilongjiang Med. 2020;44:478–81.

[59] Kang J Yang S Wang R. Clinical observation on the treatment of premature ovarian failure of spleen and kidney yang deficiency type with thermosensitive moxibustion combined with artificial cycle. Shanghai J Acupunct Moxibustion. 2021;40:715–20.

[60] Wang QW Tian Y Qiu HX. Clinical study on mild moxibustion combined with western medicine in the treatment of premature ovarian failure of spleen and kidney yang deficiency type. World J Acupunct Moxibustion. 2021;31:291–5.

[61] Zhang Y. Observation on the efficacy of Wenshen Jianpi Decoction combined with Leihuo Moxibustion in the treatment of oligomenorrhea caused by decreased ovarian reserve function. Electron J Pract Gynecol Endocrinol. 2021;8:65–8.

[62] Cui LL Kang MX Peng B. Observation on the efficacy of acupoint catgut embedding combined with Yunzhong Dingkun decoction in the treatment of premature ovarian insufficiency. Shanghai J Acupunct Moxibustion. 2022;41:573–8.

[63] Shi YG Wen HY. Observation on the effect of Yu-lin decoction combined with Du-ren moxibustion in the treatment of premature ovarian failure. Inf Tradit Chin Med. 2021;38:71–4.

[64] Cheng F Shi W Yuan F Xie X Xiang Y Chen C. Clinical observation on the efficacy of acupuncture combined with Du channel moxibustion in the treatment of premature ovarian failure of liver and kidney yin deficiency type. World J Integr Tradit West Med. 2020;15:943–945 + 950.

[65] Cao W Li N Zhao Y. Effect of acupuncture combined with Yuyin Pills on menstrual volume and serum VEGF levels in patients with decreased ovarian reserve function. J Guangzhou Univ Tradit Chin Med. 2020;37:443–7.

[66] Peng YL. Clinical observation on the treatment of premature ovarian insufficiency with acupuncture and medicine. J Pract Chin Med. 2022;38:5–6.

[67] Li Y. Clinical efficacy of acupuncture and medicine combined with estrogen-progestin sequential therapy in the treatment of kidney deficiency and blood stasis type of premature ovarian failure and its effect on pregnancy outcomes. Hubei J Tradit Chin Med. 2021;43:16–9.

[68] Li K Deng L Lu F Zhong F. Clinical study on the treatment of premature ovarian failure with Bushen Yanggan Decoction combined with acupoint catgut embedding. Chin Med Innov. 2022;19:83–7.

[69] Du J Yue A Yu W. Clinical study on the treatment of premature ovarian failure, kidney deficiency and liver depression with acupuncture therapy of Peiyuan Tiaoshen. Modern J Integr Tradit Chin West Med. 2022;31:1502–7.

[70] Li MM Su ZJ Xue BR. Effects of acupuncture combined with clomiphene on antral follicle count serum indexes and TCM syndrome scores in patients with diminished ovarian reserve. Chin J Maternal Child Health. 2022;37:4442–6.

[71] Su Z Xue B. Effects of warm acupuncture on sex hormone levels and ovarian blood flow status in patients with premature ovarian failure. Hainan Med J. 2022;33:1428–31.

[72] Wu X Fu H Li Y. Acupuncture treatment of premature ovarian failure of kidney deficiency and blood stasis type. J Tradit Chin Med. 2022;37:2452–6.

[73] Huang YF Lin J Zhong J. Effects of Bushen Huoxue Decoction combined with acupoint catgut embedding therapy on endometrial receptivity in patients with diminished ovarian reserve function. Hubei J Tradit Chin Med. 2023;45:19–22.

[74] Zhang XH Tian LJ Zhou Y. Effects of warm needling combined with umbilical moxibustion on ovarian blood flow status and reserve function in patients with premature ovarian failure of kidney yang deficiency type. Mod J Integr Tradit Chin West Med. 2023;32:935–8.

[75] Tian L Zhou Y. Effects of acupuncture on Chong and Ren meridian acupoints on ovarian responsiveness in patients with diminished ovarian reserve function. Chin J Tradit Chin Med Inf. 2023;30:167–72.

[76] Zheng Y Feng X Mi H. Effects of transcutaneous electrical acupoint stimulation on ovarian reserve of patients with diminished ovarian reserve in in vitro fertilization and embryo transfer cycles. J Obstet Gynaecol Res. 2015;41:1905–11. 26455718 10.1111/jog.12810

[77] Yang L Zhang H Zhou L. Effect of electro-acupuncture on ovarian function of women with diminished ovarian reserve: study protocol for a randomized controlled trial. Current Controlled Trials in Cardiovascular Medicine. 2021;22:921. 10.1186/s13063-021-05894-2 PMC8670117 34906206

[78] Yi A Qin X Du Z Wang T Liu F. Clinical observation on the improvement of serum sex hormone and ovarian function in premature ovarian failure patients with deficiency-cold syndrome by combining Wenjing Decoction with Tiaobu Chongren acupuncture and moxibustion. Evid Based Complement Alternat Med. 2021;2021:3926822. 34545290 10.1155/2021/3926822 PMC8449719

[79] Xu H Hao M Zheng C. Effect of acupuncture for diminished ovarian reserve: study protocol for a randomized controlled trial. Trials. 2021;22:720. 34666807 10.1186/s13063-021-05684-w PMC8527724

[80] Li HX Shi L Liang SJ. Moxibustion alleviates decreased ovarian reserve in rats by restoring the PI3K/AKT signaling pathway. J Integr Med. 2022;20:163–72. 35153135 10.1016/j.joim.2022.01.007

[81] Fan S Fang YG. Research progress of acupuncture for the improvement of ovarian reserve by regulating different signal pathways [in Chinese]. Zhen Ci Yan Jiu. 2022;47:644–8. 35880284 10.13702/j.1000-0607.20210330

